# Detrusor underactivity prevalence and risk factors according to different definitions in women attending urogynecology clinic

**DOI:** 10.1007/s00192-021-04796-w

**Published:** 2021-04-30

**Authors:** Gloria D’Alessandro, Stefania Palmieri, Alice Cola, Marta Barba, Stefano Manodoro, Matteo Frigerio

**Affiliations:** 1 Academic Unit of Obstetrics and Gynecology, IRCCS Ospedale Policlinico San Martino, Largo Rosanna Benzi, 10, 16132 Genoa, Italy; 2 University of Genoa, Genoa, Italy; 3 University of Milano-Bicocca, Monza, Italy; 4 ASST Santi Paolo e Carlo, Milan, Italy; 5 ASST Monza, Ospedale San Gerardo, Monza, Italy

**Keywords:** Detrusor underactivity, Urodynamics, Bladder contractility index, Incomplete bladder emptying, Bladder outflow obstruction

## Abstract

**Introduction and hypothesis:**

There is still no consensus on definitions of detrusor underactivity; therefore, it is difficult to estimate the prevalence. The primary objective of the study was to evaluate the prevalence of detrusor underactivity in a cohort of patients with pelvic floor disorders according to different proposed urodynamics definitions. The secondary objectives were to estimate the association between detrusor underactivity and symptoms, anatomy and urodynamic findings and to build predictive models.

**Methods:**

Patients who performed urodynamic evaluation for pelvic floor disorders between 2008 and 2016 were retrospectively analyzed. Detrusor underactivity was evaluated according to Schafer’s detrusor factor, Abrams’ bladder contractility index and Jeong cut-offs. The degree of concordance between each method was measured with Cohenʼs kappa, and differences were tested using Student’s t test, Wilcoxon test and Pearson’s chi-squared test.

**Results:**

The prevalence of detrusor underactivity among a cohort of 2092 women, concerning the three urodynamic definitions, was 33.7%, 37.0% and 4.1%, respectively. Age, menopausal status, voiding/bulging symptoms, anterior and central prolapse, first desire to void and positive postvoid residual were directly related to detrusor underactivity. Conversely, stress urinary incontinence, detrusor pressures during voiding and maximum flow were inversely associated. Final models for detrusor underactivity resulted in poor accuracy for all considered definitions.

**Conclusions:**

The prevalence of detrusor underactivity varies depending on the definition considered. Although several clinical variables resulted as independent predictors of detrusor underactivity, instrumental evaluation still plays a key role in the diagnosis.

**Supplementary Information:**

The online version contains supplementary material available at 10.1007/s00192-021-04796-w.

## Introduction

Incomplete bladder emptying may be due to bladder outflow obstruction or impaired bladder contractility, otherwise referred to as detrusor underactivity (DU) [1]. DU can result in a wide range of symptoms, including reduced flow rate or feeling of incomplete bladder emptying, storage symptoms (frequency, urgency, incontinence and nocturia) and post-voiding symptoms [1]. DU is considered to be a multifactorial condition involving the afferent side, the central nervous system and the efferent side of the micturition reflex, including the nerves and detrusor muscle [2]. Postulated causes include aging, diabetes mellitus, neurological disorders (degenerative, traumatic and infective), bladder outlet obstruction (BOO) and pelvic surgery [2]. However, the etiopathogenetic mechanisms are not well understood yet. The concept of detrusor underactivity itself remains under debate, as there is still no consensus on the terms and definitions. Different terms have been proposed, including hypotonic bladder, bladder underactivity, impaired detrusor contractility and underactive bladder [2]. The term detrusor underactivity is defined by the International Continence Society as a contraction of reduced strength and/or duration, resulting in prolonged bladder emptying and/or a failure to achieve complete bladder emptying within a normal time span [3]. However, an operative definition is not provided, and there is lack of specific urodynamic parameter thresholds [4]. Different algorithms have been proposed to evaluate and quantify detrusor contractility including Schafer’s detrusor factor [5], Abrams’ bladder contractility index [6] and the use of specific cut-offs for maximum flow (Qmax) and detrusor pressure maximum flow (Pdet@Qmax) [7]. Unfortunately, none of these have been validated in the female population [8]. Since the diagnostic criteria are not well defined, it is difficult to estimate the prevalence of DU in the female population. In patients bothered by lower urinary tract symptoms, DU can be identified in about 13% of women [7]. However, in specific populations, such as in women with advanced pelvic organ prolapse, the prevalence may increase up to 40% [9].

The primary objective of the study was to evaluate the prevalence of DU in a cohort of patients with pelvic floor disorders according to different proposed urodynamics definitions. The secondary objectives were to estimate the association between DU diagnosis and symptoms, anatomy and urodynamic findings and to build predictive models.

## Materials and methods

Patients who underwent primary urodynamics evaluation for pelvic floor disorders between 2008 and 2016 were retrospectively analyzed. Clinical evaluation included a medical interview to investigate the presence of urinary and prolapse symptoms. A urogenital examination was performed, and pelvic organ prolapse was staged according to the Pelvic Organ Prolapse Quantification (POP-Q) system. All women underwent urodynamic assessment as previously described (including filling cystometry, pressure/flow study and post-void residual (PVR) volume by catheterization) by a trained urogynecologist [10]. Procedures and definitions conformed to the Good Urodynamic Practice Guidelines of the International Continence Society [11]. Positive PVR was defined as a residual > 100 ml. Detrusor underactivity was evaluated using three different methods: Schafer’s detrusor factor [5], Abrams’ bladder contractility index [6] and Jeong et al.'s cut-offs [7]. DU based on Schafer nomogram was defined for values of Qmax and Pdet@Qmax ranging from very weak (VW) and weak- (W-) to weak+ (W+) according to detrusor contractility and from zone 0 and I according to urinary obstruction (DU). The bladder contractility index (BCI = pDetQmax + Qmax × 5) was also evaluated to analyze detrusor contractility. Specifically, a BCI < 100 was considered indicative of a DU according to Abrams’ definition (DU_A_). Lastly, cut-offs of Qmax ≤ 12 and Pdet@Qmax10 were applied to define DU according to Jeong et al.'s definition (DU_J_). For every definition applied, non-DU patients were considered as controls.

The study was approved by the Ethics Committee of San Gerardo Hospital in Monza, Italy. Data were entered into the database by one author and double-checked by another author. Statistical analysis was performed using JMP software version 9.0 (SAS, Cary, NC, USA). Data are reported as mean ± standard deviation. The degree of concordance/agreement between the different definitions of DU was measured with Cohenʼs kappa [12]. Differences were tested using Student’s *t*-test for continuous parametric data, the Wilcoxon test for continuous nonparametric data and Pearson’s chi-squared test for noncontinuous data. A *p* value < 0.05 was considered statistically significant. Multivariate models to predict DU according to the considered definitions were built using variables from population characteristics, referred symptoms and clinical examinations (thus excluding urodynamic parameters). Only variables significantly associated with the outcomes in the univariate analysis were considered, excluding the ones with possible collinearity. Odds ratios were provided to evaluate the strength of association for continuous data and non-continuous data, respectively.

## Results

**Fig. 1:**
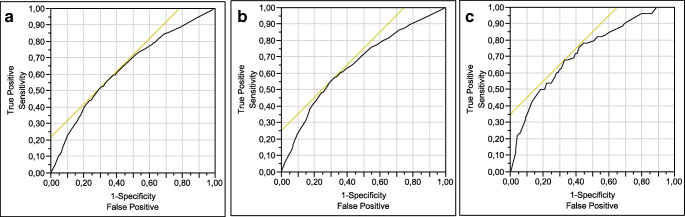
Receiver-operating characteristics curve for DU_S_ (**a**), DU_A_ (**b**) and DU_J_ (**c**). Y-axis: sensitivity; X-axis: 1-specificity

A total of 2092 women underwent urodynamic evaluation in the study period. Full records were available for 1972 (exclusion rate of 5.7%). Fifty-one patients were not able to start micturition because of severe voiding dysfunction. As a consequence, pressure/flow study parameters were available for the remaining 1921 patients. Patients’ characteristics and symptoms leading to urodynamic evaluation are reported in Table 1. The mean age was 61.0 ± 12.8 years. The most frequently reported symptom was stress urinary incontinence (61.6%), followed by overactive bladder syndrome (57.5%). Voiding symptoms and vaginal bulging were reported by 35.6% and 42.1% of patients, respectively. The anterior vaginal compartment was the one most frequently involved in a significant (≥ 2 stage) descensus. According to the considered definitions, prevalence of DU_J_, DU_S_ and DU_A_ was 4.1%, 33.7% and 37.0%, respectively. An excellent degree of agreement was found between DU_S_ and DU_A_ (k = 0.93), while DU_J_ showed a very poor concordance with both DU_S_ (k = 0.15) and DU_A_ (k = 0.13). Univariate analysis of risk factors for DU according to the different definitions is reported in Supplementary Table 1. At the univariate analysis, age, menopausal status, voiding symptoms, bulging symptoms, anterior and central compartment prolapse stage ≥ 2, first desire to void and positive postvoid residual were directly related to detrusor underactivity for all considered definitions. Conversely, stress urinary incontinence and detrusor pressures during voiding and maximum flow were inversely associated with detrusor underactivity for all considered definitions. Urodynamic stress incontinence was a protective factor for both DU_S_ and DU_A_. Higher cystometric capacity resulted as a protective risk factor for DU_S_ and DU_A_, while it was directly associated with DU_J_. The latter was also associated with less overactive bladder syndrome and detrusor overactivity. On the multivariate analysis, age (OR = 1.9–2.3) and voiding symptoms (OR = 1.7–2.5) were independent predictors of DU according to all considered definitions (Table 2). Stress urinary incontinence was a protective factor towards both DU_A_ and DU_S_ (OR = 0.7–0.8), while overactive bladder syndrome was protective toward DU_J_ (OR = 0.6). The final models for DU had poor accuracy for all considered definitions, with areas under the curve (AUC) ranging between 0.64 and 0.72 (Fig. 1).

**Table 1:** Population characteristics. Continuous data as mean ± standard deviation. Non-continuous data as absolute frequency (relative frequency)

Age (years)	61.0 ± 12.8
Body mass index (kg/m^2^)	26.5 ± 4.7
Parity (*n*)	1.9 ± 1.2
Instrumental delivery	183 (9.3%)
Maximal birth weight (g)	3479 ± 702
Menopausal status	1580 (80.1%)
Overactive bladder syndrome	1134 (57.5%)
Stress urinary incontinence	1215 (61.6%)
Voiding symptoms	703 (35.6%)
Bulging symptoms	817 (42.1%)
Anterior prolapse stage ≥ 2	855 (43.4%)
Central prolapse stage ≥ 2	512 (26.0%)
Posterior prolapse stage ≥ 2	476 (24.1%)

**Table 2:** Multivariate analysis. Parameter estimate and odds ratio are provided for continuous data and non-continuous data, respectively

	DU_S_		DU_A_		DU_J_	
	OR	*p* value	OR	*p* value	OR	*p* value
Age ≥ 65 years	1.9	< 0.0001	1.9	< 0.0001	2.3	0.0008
Stress urinary incontinence	0.8	0.0217	0.7	0.0008	0.7	0.15
Overactive bladder syndrome	1.0	0.95	1.0	0.76	0.6	0.0214
Voiding symptoms	1.7	< 0.0001	1.8	< 0.0001	2.5	0.0002
Bulging symptoms	1.2	0.11	1.2	0.27	0.9	0.72
Anterior stage prolapse ≥ 2	1.0	0.81	1.2	0.25	1.6	0.18

## Discussion

DU probably represents an underestimated condition, which may involve voiding dysfunction and long-term sequelae. To date, the prevalence of DU in the female population with pelvic floor disorders is not defined. With this study, we aimed to evaluate the prevalence of DU in a cohort of patients with pelvic floor disorders according to three different proposed urodynamics definitions and to evaluate the association among DU diagnosis and symptoms, anatomy and urodynamic findings. We found that the prevalence of DU can greatly vary depending on the definition considered, ranging from 4.1% to 37.0% in a population of women afferent to pelvic floor clinics. DU was associated with specific urodynamic findings compared to controls, including an increased first desire to void, lower detrusor pressures during voiding and maximum flow, and a higher rate of positive post-void residuals. Moreover, an inconsistent association was found for the maximum cystometric capacity and the diagnosis of urodynamic stress incontinence and detrusor overactivity. The multivariate analysis demonstrated that age > 65 years, the lack of stress urinary incontinence and the presence of voiding symptoms are independent predictors of DU. However, models to predict DU based on variables from population characteristics, baseline symptoms and clinical examination showed poor accuracy, indicating the key role of urodynamics in identifying this condition in case of suspicion.

The great heterogeneity of DU prevalence reported in the literature is likely to depend on population characteristics such as sex, age and reasons to perform urodynamics—and on the definitions applied. A previous experience reported a 13% rate of DU in women bothered by lower urinary tract symptoms [7]. However, Jeong et al. looked at the concordance of urodynamic criteria in the diagnosis of DU in a population of 372 patients, finding significant variations in the prevalence using the different criteria, ranging from 5.4% to 55.8% [13]. Similarly, in our population, we found a prevalence ranging from 4.1% to 37.0% according to the different definitions considered. Specifically, we demonstrated a substantial agreement between the definitions of Schafer and Abrams, with the latter probably slightly overestimating the prevalence due to lack of bladder outlet resistance evaluation, thus classifying a portion of patients with BOO as DU. Conversely, Jeong’s definition is likely to identify the most neglected and severe forms of DU, but at the cost of a lower detection rate.

From a urodynamic point of view, DU is characterized by low flow and detrusor pressures during voiding, which are the two parameters used to estimate detrusor strength [14]. This might lead to impaired voiding, with significant post-void residuals. In our study, positive PVR was found four to nine times more frequently in patients with DU—depending on the definition considered—compared to controls. This confirms the great impact of impaired bladder contractility as a cause or contributing factor in voiding dysfunctions. However, we identified additional urodynamic findings associated with DU. The first desire to void was registered at a significantly higher volume in patients with DU compared to controls. This finding suggests a major role of disruption of the bladder innervation in the pathogenesis of DU, which may be reflected in an impairment of the sensory afferent pathways. This mechanism has already been proposed for the loss of bladder contractility related to diabetic cystopathy, in which an autonomic neuropathy and a diminished bladder sensation, resulting from the axonal degeneration and the segmental demyelination, have been demonstrated [15]. Probably, both the afferent and efferent compartments of the micturition reflex are equally involved in the pathogenesis of detrusor underactivity, determining both sensory and detrusor muscle function impairment. We also found a lower prevalence of urodynamic stress urinary incontinence in DU_S_ and DU_A_ but not DU_J_ patients. Both DU_S_ and DU_A_ definitions are likely to be less specific compared to DU_J_ and might also include patients with a certain grade of BOO. As a consequence, it is reasonable to find a lower prevalence of urodynamic stress incontinence, which can be considered as surrogate marker of lower urethral resistance. Conversely, the DU_J_ definition is very restrictive and likely to exclude patients with BOO, identifying women with a more advanced stage of detrusor impairment. This may be the explanation for the different findings regarding the maximum cystometric capacity and detrusor overactivity in DU_J_ patients compared to both DU_S_ and DU_A_ women. In fact, we recorded a lower prevalence of detrusor overactivity and a higher maximum cystometric capacity only in DU_J_ patients. These findings can be associated with a pronounced alteration of bladder sensitivity and impairment of detrusor contractile power, which are demonstrated mechanisms of DU [16, 17]. Moreover, neglected DU with chronic urinary retention may lead to increased bladder volumes through progressive stretching/thinning of the bladder wall [18, 19].

Interestingly, the decrease in sensory function and muscle contractility has also been described as a consequence of normal bladder aging [20]. Our study confirmed the role of aging as a risk factor for detrusor underactivity, irrespective of the diagnostic criterion considered. In particular, age ≥ 65 years was found to be an independent predictor of DU, with an odds ratio ranging from 1.9 to 2.3 according to the considered definition. Different explanations have been proposed. Specifically, an age-dependent reduction in the amount of acetylcholinesterase-positive nerves has been reported, suggesting a reduced functional parasympathetic innervation. Moreover, a muscle-to-collagen ratio reduction and decrease in axonal content have been described as possible contributors to the decline in sensory and muscle functions in the elderly [20].

Our study also confirmed the predictive role of the voiding symptoms, which carry a 1.7–2.5 times greater risk of being associated with DU. Voiding symptoms such as “prolonged urination time with or without a sensation of incomplete bladder emptying, usually with hesitancy, reduced sensation on filling, and a slow stream” are known to be suggestive of DU, according to the underactive bladder working definition [21]. Our findings are concordant with a previous urodynamic study on 1788 women and men, in which patients with DU had significantly higher occurrence of decreased and/or interrupted urinary stream, hesitancy, feeling of incomplete bladder emptying and absent and/or decreased sensation compared with controls [22]. The prevalent voiding disorder nature associated with DU diagnosis was also confirmed by the protective role of the storage symptoms, such as stress urinary incontinence (for DU_S_ and DU_A_) and overactive bladder syndrome (for DU_J_) found in our population. The rationales are supposed to be similar those previously proposed for urodynamic stress urinary incontinence and overactive bladder. The first can be considered a surrogate marker of lower urethral resistance, thus indicating a portion of the population in which the prevalence of voiding disorders—including DU—is less pronounced. The latter is likely to be associated with maintained/increased detrusor muscle contractility, thus an opposite mechanism to the one proposed in DU etiopathogenesis.

However, despite DU being a predominantly voiding disorder, no milestone symptom characterizes underactivity of the detrusor. In our series, patients with DU reported either voiding symptoms or storage symptoms, or both. The consequence was that multivariate models built on non-instrumental variables failed to identify patients with DU with good accuracy, for all considered definitions, with AUC ranging from 0.64 to 0.72. This also confirms for DU that “the bladder is an unreliable witness of itself.” Considering the considerable overlapping and the nonspecific nature of symptoms, it is not possible to reliably differentiate DU from other lower urinary tract dysfunctions without a urodynamic study [21]. As a consequence, in case of symptoms evocative for DU—such as the ones identified as risk factors in our study (e.g., voiding sympoms, bulging symptoms)—a urodynamic evaluation is recommended. However, our study clearly demonstrated the urgent need to provide a “urodynamic” definition to correctly diagnose detrusor underactivity, since the range of prevalence based on different definitions is too wide to provide clinical guidance. In our opinion, the best choice is the use of the Schafer nomogram, which offers some advantages compared to the other two considered definitions. It allows discriminating DU from BOO, which is not possible using Abrams’ bladder contractility index. Moreover, the use of dynamic thresholds compared to the specific ones proposed by Jeong allows identifying a larger number of patients with DU.

The strengths of this study include the large population considered, multimodal evaluation of patients with anthropometric characteristics, and the presence of obstetric history, baseline symptoms, the gynecological visit and urodynamics as well as the application of three different definitions of detrusor underactivity. The limitations include the retrospective design and the lack of Griffiths' watt factor evaluation.

To conclude, the prevalence of DU can vary greatly depending on the definition considered, ranging from 4.1% to 37.0% in a population of women attending our pelvic floor clinics. Age > 65 years, lack of stress urinary incontinence and the presence of voiding symptoms are independent predictors of DU. However, models to predict DU based on clinical variables are inaccurate, indicating the key role of instrumental evaluation in identifying this condition.

Future research should focus on the standardization and validation of urodynamics parameters in women based definitions.

## Supplementary Information

ESM 1 (DOC 82 kb)

## References

[1] Aldamanhori R, Chapple CR (2017). Underactive bladder, detrusor underactivity, definition, symptoms, epidemiology, etiopathogenesis, and risk factors. Curr Opin Urol.

[2] Andersson KE (2014). Bladder underactivity. Eur Urol.

[3] Abrams P, Cardozo L, Fall M, Griffiths D, Rosier P, Ulmsten U (2002). The standardisation of terminology of lower urinary tract function: report from the standardisation sub-committee of the international continence society. Neurourol Urodyn.

[4] D'Ancona C, Haylen B, Oelke M, Abranches-Monteiro L, Arnold E, Goldman H, Hamid R, Homma Y, Marcelissen T, Rademakers K, Schizas A, Singla A, Soto I, Tse V, de Wachter S, Herschorn S, Standardisation Steering Committee ICS, the ICS Working Group on Terminology for Male Lower Urinary Tract & Pelvic Floor Symptoms and Dysfunction (2019). The International Continence Society (ICS) report on the terminology for adult male lower urinary tract and pelvic floor symptoms and dysfunction. Neurourol Urodyn.

[5] Schafer W (1995). Analysis of bladder-outlet function with the linearized passive urethral resistance relation, linPURR, and a disease-specific approach for grading obstruction: from complex to simple. World J Urol.

[6] Abrams P (1999). Bladder outlet obstruction index, bladder contractility index and bladder voiding efficiency: three simple indices to define bladder voiding function. BJU Int.

[7] Jeong SJ, Kim HJ, Lee YJ, Lee JK, Lee BK, Choo YM (2012). Prevalence and clinical features of detrusor underactivity among elderly with lower urinary tract symptoms: a comparison between men and women. Korean J Urol.

[8] Van Koeveringe GA, Vahabi B, Andersson KE, Kirschner-Herrmans R, Oelke M (2011). Detrusor underactivity: a plea for new approaches to a common bladder dysfunction. Neurourol Urodyn.

[9] Frigerio M, Manodoro S, Cola A, Palmieri S, Spelzini F, Milani R (2018). Detrusor underactivity in pelvic organ prolapse. Int Urogynecol J.

[10] Manodoro S, Spelzini F, Frigerio M, Nicoli E, Verri D, Milani R (2016). Is occult stress urinary incontinence a reliable predictive marker?. Female Pelvic Med Reconstr Surg.

[11] Abrams P, Andersson KE, Birder L, Brubaker L, Cardozo L, Chapple C (2010). Fourth international consultation on incontinence recommendations of the international scientific Committee: evaluation and treatment of urinary incontinence, pelvic organ prolapse, and fecal incontinence. Neurourol Urodyn.

[12] Koch GG, Landis JR, Freeman JL (1977). A general methodology for the analysis of experiments with repeated measurement of categorical data. Biometrics.

[13] Jeong SJ, Lee JK, Kim KM, Kook H, Cho SY, Oh SJ (2017). How do we diagnose detrusor underactivity? Comparison of diagnostic criteria based on an urodynamic measure. Invest Clin Urol.

[14] Griffiths DJ (2003). Editorial: bladder failure – a condition to reckon with. J Urol.

[15] Hill SR, Fayyad AM, Jones GR (2008). Diabetes mellitus and female lower urinary tract symptoms: a review. Neurourol Urodyn.

[16] Osman NI, Chapple CR (2014). Contemporary concepts in the aetiopathogenesis of detrusor underactivity. Nat Rev Urol.

[17] Brierly RD, Hindley RG, McLarty E (2003). A prospective controlled quantitative study of ultrastructural changes in the underactive detrusor. J Urol.

[18] Makar AA, Thomas PJ, Fletcher MS, Harrison NW (1998). Transballoon cystometry: a new technique to assess detrusor function after urinary retention. Br J Urol.

[19] Rademakers KL, van Koeveringe GA, Oelke M, FORCE Research Group, Maastricht and Hannover (2017). Ultrasound detrusor wall thickness measurement in combination with bladder capacity can safely detect detrusor underactivity in adult men. World J Urol.

[20] Gilpin SA, Gilpin CJ, Dixon JS (1986). The effect of age on the autonomic innervation of the urinary bladder. Br J Urol.

[21] Chapple CR, Osman NI, Birder L (2015). The underactive bladder: a new clinical concept?. Eur Urol.

[22] Gammie A, Kaper M, Dorrepaal C, Kos T, Abrams P (2016). Signs and symptoms of detrusor underactivity: an analysis of clinical presentation and urodynamic tests from a large Group of Patients Undergoing Pressure Flow Studies. Eur Urol.

