# Supplementary material for: Detrusor underactivity prevalence and risk factors according to different definitions in women attending urogynecology clinic
Source: Int Urogynecol J. 2021 Apr 30;33(4):835–40. doi: 10.1007/s00192-021-04796-w (PMC9021137; doi:10.1007/s00192-021-04796-w)
Supplement: Supplementary file 1 — (DOC 82 kb) [file 192_2021_4796_MOESM1_ESM.doc]

**Supplementary Table 1**

Univariate analysis of risk factors for DU according to the different definitions. Continuous data as mean ± standard deviation. Non-continuous data as absolute frequency (relative frequency).

|  | | | DU_S_ | | | | | DU_A_ | | | | | DU_J_ | | |
| --- | --- | --- | --- | --- | --- | --- | --- | --- | --- | --- | --- | --- | --- | --- | --- |
|  |  |  | Mean / Freq | | p | | Mean / Freq | | | p | | Mean / Freq | | | p |
| Age (years) | DU | 64.0 ± 12.2 | | <.0001 | | 63.9 ± 12.2 | | | <.0001 | | 67.0 ± 14.0 | | | .0015 | |
|  | Controls | 59.5 ± 12.8 | |  |  | 59.3 ± 12.8 | | |  |  | 60.8 ± 12.7 | | |  |  |
| Body Mass Index (kg/m^2^) | DU | 26.5 ± 4.6 | | .82 | | 26.4 ± 4.6 | | | .86 | | 26.0 ± 4.6 | | | .34 | |
|  | Controls | 26.6 ± 4.7 | |  |  | 26.6 ± 4.7 | | |  |  | 26.6 ± 4.7 | | |  |  |
| Parity (n) | DU | 1.9 ± 1.2 | | .34 | | 1.9 ± 1.2 | | | .20 | | 1.8 ± 1.2 | | | .58 | |
|  | Controls | 1.9 ± 1.1 | |  |  | 1.9 ± 1.1 | | |  |  | 1.9 ± 1.2 | | |  |  |
| Instrumental delivery | DU | 59 (8.9%) | | .67 | | 64 (8.8%) | | | .56 | | 6 (7.5%) | | | .58 | |
|  | Controls | 124 (9.5%) | |  |  | 119 (9.6%) | | |  |  | 177 (9.4%) | | |  |  |
| Maximal birth-weight (g) | DU | 3538 ± 690 | | .16 | | 3531 ± 676 | | | .19 | | 3496 ± 731 | | | .40 | |
|  | Controls | 3454 ± 706 | |  |  | 3453 ± 713 | | |  |  | 3479 ± 701 | | |  |  |
| Menopausal status | DU | 571 (86.0%) | | <.0001 | | 626 (85.9%) | | | <.0001 | | 71 (88.8%) | | | .0484 | |
|  | Controls | 1009 (77.1%) | |  |  | 954 (76.8%) | | |  |  | 1509 (79.8%) | | |  |  |
| Overactive bladder syndrome | DU | 378 (56.9%) | | .71 | | 417 (57.2%) | | | .83 | | 34 (42.5%) | | | .0056 | |
|  | Controls | 576 (57.8%) | |  |  | 717 (57.7%) | | |  |  | 1100  (58.1%) | | |  |  |
| Stress urinary incontinence | DU | 353 (53.2%) | | <.0001 | | 377 (51.7%) | | | <.0001 | | 32 (40.0%) | | | <.0001 | |
|  | Controls | 862 (65.9%) | |  |  | 838 (67.4%) | | |  |  | 1183 (62.5%) | | |  |  |
| Voiding symptoms | DU | 302 (45.5%) | | <.0001 | | 338 (46.4%) | | | <.0001 | | 50 (62.5%) | | | <.0001 | |
|  | Controls | 401 (30.7%) | |  |  | 365 (29.4%) | | |  |  | 653 (34.5%) | | |  |  |
| Bulging symptoms | DU | 325 (49.7%) | | <.0001 | | 359 (50.1%) | | | <.0001 | | 45 (57.7%) | | | .0043 | |
|  | Controls | 492 (38.2%) | |  |  | 458 (37.4%) | | |  |  | 772 (41.4%) | | |  |  |
| Anterior prolapse stage ≥ 2 | DU | 328 (49.4%) | | .0001 | | 370 (50.8%) | | | <.0001 | | 48 (60.0%) | | | .0022 | |
|  | Controls | 527 (40.3%) | |  |  | 485 (39.0%) | | |  |  | 807 (42.7%) | | |  |  |
| Central prolapse stage ≥ 2 | DU | 221 (33.3%) | | <.0001 | | 250 (34.3%) | | | <.0001 | | 32 (40.0%) | | | .0035 | |
|  | Controls | 291 (22.3%) | |  |  | 262 (21.1%) | | |  |  | 480 (25.4%) | | |  |  |
| Posterior prolapse stage ≥ 2 | DU | 161 (24.3%) | | .94 | | 181 (24.8%) | | | .58 | | 24 (30.0%) | | | .21 | |
|  | Controls | 315 (24.1%) | |  |  | 295 (23.7%) | | |  |  | 452 (23.9%) | | |  |  |
| First desire to void (ml) | DU | 159 ± 80 | | .0386 | | 160 ± 80 | | | .0093 | | 199 ± 92 | | | <.0001 | |
|  | Controls | 153 ± 82 | |  |  | 152 ± 82 | | |  |  | 153 ± 80 | | |  |  |
| Maximum cystometric capacity (ml) | DU | 386 ± 100 | | .0008 | | 387 ± 99 | | | .0011 | | 428 ± 101 | | | .0040 | |
|  | Controls | 401 ± 99 | |  |  | 401 ± 99 | | |  |  | 394 ± 99 | | |  |  |
| Opening detrusor pressure (cmH2O) | DU | 17.8 ± 10.7 | | <.0001 | | 19.8 ± 13.1 | | | 0.17 | | 4.6 ± 7.9 | | | <.0001 | |
|  | Controls | 22.1 ± 14.8 | |  |  | 21.1 ± 14.1 | | |  |  | 21.3 ± 13.5 | | |  |  |
| Closure detrusor pressure (cmH2O) | DU | 18.5 ± 13.3 | | <.0001 | | 20.3 ± 14.6 | | | .0010 | | 2.8 ± 4.6 | | | <.0001 | |
|  | Controls | 24.1 ± 17.0 | |  |  | 23.3 ± 16.7 | | |  |  | 23.0 ± 15.8 | | |  |  |
| Detrusor pressure at peak (cmH2O) | DU | 20.0 ± 10.6 | | <.0001 | | 22.5 ± 13.2 | | | <.0001 | | 3.7 ± 4.7 | | | <.0001 | |
|  | Controls | 27.9 ± 19.8 | |  |  | 26.8 ± 19.7 | | |  |  | 26.2 ± 17.4 | | |  |  |
| Maximum flow (ml/s) | DU | 10.2 ± 4.5 | | <.0001 | | 9.8 ± 4.5 | | | <.0001 | | 2.7 ± 3.6 | | | <.0001 | |
|  | Controls | 23.1 ± 9.1 | |  |  | 24.0 ± 8.4 | | |  |  | 19.4 ± 9.5 | | |  |  |
| Positive post-void residual | DU | 193 (29.1%) | | <.0001 | | 236 (32.4%) | | | <.0001 | | 63 (78.8%) | | | <.0001 | |
|  | Controls | 87 (6.7%) | |  |  | 44  (3.5%) | | |  |  | 217 (11.5%) | | |  |  |
| Urodynamic stress urinary incontinence | DU | 278 (41.9%) | | .0003 | | 298 (40.9%) | | | <.0001 | | 31 (38.8%) | | | 0.11 | |
|  | Controls | 661 (50.5%) | |  |  | 641 (51.6%) | | |  |  | 908 (48.8%) | | |  |  |
| Detrusor overactivity | DU | 220 (33.1%) | | .82 | | 244 (33.5%) | | | >0.99 | | 11 (13.8%) | | | .0001 | |
|  | Controls | 440 (33.6%) | |  |  | 416 (33.5%) | | |  |  | 649 (34.3%) | | |  |  |
